# Sociality, resilience and agency: how did young Australians experience online learning during Covid-19?

**DOI:** 10.1007/s13384-021-00500-5

**Published:** 2021-12-17

**Authors:** Loshini Naidoo, Jacqueline D’warte, Susanne Gannon, Rachael Jacobs

**Affiliations:** 1 School of Education, Western Sydney University, Second Ave, Kingswood, 2747 Australia; 2 School of Education, Western Sydney University, Bankstown Campus, Milperra, 2214 Australia

**Keywords:** Student voice, Agency, Resilience, Educational futures, Online learning

## Abstract

In 2020 when schooling was abruptly reconfigured by the pandemic, young people were required to demonstrate new capabilities to manage their learning and their wellbeing. This paper reports on the feelings, thoughts and experiences of eight Year 9 and 10 students in NSW and Victoria about the initial period of online learning in Australian schools that resulted from the Covid-19 pandemic. Beyond dominant narratives of vulnerability and losses in learning, our participants offered counternarratives that stressed their capacities to rise and meet the times. We trace three central themes on how they: found moments of agency that increased their confidence, reconfigured resilience as a socially responsible set of practices, deployed sociality as a resource for the benefit of themselves and others. The pandemic opened up conversations with young people about where and how learning takes place and how schools might adapt and respond to young people’s growing sense of urgency about the future of schooling.

## Introduction

For education systems around the world, the long Covid-19 pandemic continues to prompt rethinking of where and how learning takes place, and of what matters most. Research is emerging about losses in learning, the effects on young people’s mental health, and effects on teachers and schools. Uneven impacts have been nuanced by national and regional responses and policy settings, the relative robustness of institutions, geography, human behaviours and many other factors, not least being the vigour and ingenuity of the virus itself.

On a global scale, Australia’s experience has at times seemed quite different from most other countries with the advantage of geographic isolation and imposed border closures keeping infections and fatalities lower that most other parts of the world. When we began our research in mid-2020, all states and educational systems had imposed extended school closures at approximately the same time,^1^ flipping to online learning for most of Term 2, 2020. Extended closures have been experienced in Victoria and NSW, with sporadic circuit-breaking closures in other states and locations as required. Concerns about the negative effects of disrupted schooling have underpinned mental health and wellbeing initiatives and state-wide tutoring interventions in several jurisdictions. While vulnerability is undoubtedly the dominant narrative about the pandemic, we are interested in this paper in glimmers of hope and reconfigured notions of resilience that young people offered as they reflected on the Covid present experiences and post-pandemic futures. A growing sense of urgency about the future of schooling and a reimagining of education systems makes privileging the perspectives of young people particularly important. Yet voices of young people have rarely been heard during the Covid-19 pandemic (Wang et al., 2020).

The young people’s responses in this study drew our attention to issues that were rarely addressed in public commentary and forced us to rethink how young people have deployed resources for learning and social and emotional support in ways that may not be recognised by adults. Our contention is that, when schooling was abruptly reconfigured by Covid-19, young people demonstrated agentic capacities that can redefine resilience and point towards some of the changes that schools might take forward into a more desirable future. In the following section we outline emerging perspectives on online schooling during Covid-19 and the conceptual resources that we work with through the paper.

## Young people, agency, resilience, sociality and learning in pandemic times

Social isolation was a key concern for many young people (yourtown & Australian Human Rights Commission, 2020). At an individual level, young people suddenly lost many of the activities that provide structure, meaning, and daily rhythms to their lives, such as school, extra-curricular activities, social interactions, and physical activity (Courtney et al., 2020).

However, glimpses of the agency and creativity of young people have also been illuminated by the pandemic. Despite challenging conditions, some students did well during the lockdown and may even have thrived in this environment (Thakur, 2020).

Agency—understood broadly as the capacity to act or to create change in the world—is an essential element of democratic participation; however, it emerges in precise and situated contexts, it is contingent and constrained by diverse material and discursive conditions. For young people, whose lives are highly regulated by the institutions and practices of schooling (such as mandated curriculum, testing, timetables, uniforms, rules and so on) opportunities to exercise agency are quite limited. Within schools, student agency is often attached to student voice initiatives which are unevenly distributed, favouring those already privileged by race and class, articulate, middle class and older students who are assumed to be able to ‘ventriloquise’ the experiences of ordinary students (Finneran et al., 2021, p. 7). Although there has been considerable educational research into student voice, aiming to encourage input and promote student-led reform, that is to enhance students’ agency within educational contexts, student voices are often co-opted or marginalised, without the extent of authentic sharing of responsibility and codesign that would lead to pedagogic change (Mockler & Groundwater-Smith, 2015), or reduced to “mere compliance” within existing structures of schooling (Charteris & Thomas, 2017, p. 163).

In pandemic times, among the accounts of ‘lost’ learning there are also some indications emerging of greater scope for student agency. In England, some students transitioning from primary to secondary schools during lockdown reported that the greater autonomy they experienced enabled them to develop and pursue their own learning interests (Leaton-Gray et al., 2021). As Biesta (2020, p. 1) points out, “learning didn’t stop” with young people learning “all kinds of useful things”, including lessons about inequality, though it is unclear how or whether schools connected productively with that learning. In the United States, schools that have promoted authentic and culturally responsive learning—where agency, identity and voice are central—are most successful in keeping students motivated and participating in learning (Darling-Hammond et al., 2020). Creative engagements with pandemic experiences have also emerged in Australian schools (Gannon et al. 2021). Digital media has also provided opportunities for young people to claim agency that may otherwise not have been open to them, enabling their voices to be heard and stimulating collective action for the wellbeing of their societies.

Resilience has been widely acknowledged as a necessary skill or quality for transitioning to life after the COVID-19 pandemic (OECD 2020b; Schwartzman, 2020). Current definitions of resilience have been influenced by views of resilience as a personality trait, and views of resilience as a process. In the former, resilience is viewed as a stable personality trait and therefore underestimates the impact of contextual and external factors (Ungar, 2012). Process definitions focus on factors that enable a positive outcome in the wake of adversity. For example, resilience is the “capacity to rebound from adversity strengthened and more resourceful” (Walsh, 2006, p. 4), or “the potential or manifested capacity of a dynamic system to adapt successfully to disturbances that threaten the function, survival, or development of the system” (Masten, 2015, p. 187) or “the process of adjusting well to significant adversity” (Theron, 2016, p. 636). In its emphasis on regaining stability after disruption, and its focus on the individual, conventional views of resilience are “fundamentally conservative” (Third et al., 2019, p. 61). To broaden its conceptual scope, Ungar (2008, p. 225) offers a “socio-ecological” definition of resilience:

In the context of exposure to significant adversity, resilience is both the capacity of individuals to navigate the psychological, social, cultural and physical resources that sustain their wellbeing, and their capacity individually and collectively to negotiate for these resources to be provided and experienced in culturally meaningful ways.

Thus, resilience is a dynamic process that emerges through the experience of adversity at both individual and collective levels. By including “culturally meaningful ways” into the definition, Ungar (2008) acknowledges the importance of culture, context and lived experience showing that both adversity and resilience are social constructions. Resilience emerges within broader sociocultural and physical ecologies; it is relational as much as it is individualised. In education, collective understandings of resilience, including their relational dimensions, have great potential. It is important to remember however, that resilience manifests in varied ways. Young people who are marginalised and socially excluded may deploy non-normative or alternative coping skills as a form of “hidden resilience” (Ungar, 2008).

Much resilience literature on how individuals transcend social risks suggests an alignment of resilience with neoliberalism. Young people must not only “bounce back from adversity” but also “bounce forward” to benefit businesses, communities and national economies (GBCE, 2020, p. 9). In neoliberalism, as Van Breda (2018) points out, accountability devolves to the individual who is made responsible for improving their life context with minimal support from the state and the erasure of attention to poverty, racism, uneven resourcing and educational inequality. Other critical perspectives argue for attention to both “structural contexts” and “reflexive agency” as social structures are always themselves being reconfigured (Estêvão et al., 2017, p. 17). From this perspective, resilience processes entail both mobilisation of resources and shifting of risks. Resources can be economic, social, cultural and environmental and include kinship and friendship as social resources. They vary in terms of how effectively they are used and are always impacted by inequalities and power imbalances. Schwartzman (2020) outlines a potential pedagogy of socially responsible resilience that would build strength as it fosters endurance, adaptability, and resistance. His “pandemic pedagogy” would take up the opportunity for renewal that the pandemic offers to cultivate three forms of resilience: (i) as *endurance*, where resilience would allow acknowledgement of limitations, and cultivate perseverance in the face of adversity; (ii) as *adaptability*, where resilience would build tolerance for uncertainty, and promote flexibility and creativity; and (iii) as *resistance*, where resilience would demand advocacy to counteract oppressions and inequities (Schwartzman, 2020, pp. 512–513).

In situations of rapid change, such as the current pandemic, young people need a sense of stability so that they can think through, adapt, and develop new strategies for coping with “emerging and fluid contexts” (Drane et al., 2020). Having a safe and secure home, and positive relationships with caring adults and peers helps young people develop their capacity to be resilient. While stay-at-home measures limited the spread of the virus, they increased feelings of loneliness and social isolation, but also inadvertently provided the impetus for young people to find creative ways to stay connected with others. The new social norms limited young people’s interactions with teachers and peers, with potential mental health-related consequences (Larcher et al., 2020, p. 1192). School connectedness, that is how staff care for students, their safety, happiness and connection to learning, is associated with motivation, academic improvement, and positive physical and mental health outcomes (Cahill et al., 2014). Much effort was invested by schools and teachers in maintaining relationships with their students as a connection to the school also contributes to young people’s resilience (Angelkovski, 2016).

Sociality is commonly viewed as an individual’s social practices, their social participation, interaction and cultivation of wide-ranging relationships (LaMendola, 2010). Sociality was at the centre of our participants’ responses, and new thinking about sociality more closely embraces our rapidly changing communicative landscape. Increasingly, cross disciplinary conceptualisations of sociality in sociocultural anthropology, sociology and post humanist theory, while once variable, come together to foreground dynamism and the agentive possibilities that emerge from co-production, flexibility, transformation and experimentation inherent in our evolving multimodal communicative pathways (Long & Moore, 2012).

In our analysis we considered participants’ expressed views of the ways sociality was both restricted and enhanced by evolving communicative technologies. We position sociality as going beyond how people interact to consider space and distance, and the way these young people related to others through action.

## Research design and method

Our research into young people’s experiences of school lockdowns in Australia parallels a study initiated by Søndergaard and colleagues from Aarhus University, Denmark (Hansen et al., 2020). Both studies were interested in seeking the voices of young people about the time when their secondary schools abruptly closed. Ethical approval to conduct the research was gained through Western Sydney University’s Human Research Ethics Approval process. The design included an anonymous online survey, interviews (via Zoom) and artefacts responding to the pandemic (Gannon et al. 2021). Information about the study was circulated on Facebook and through our networks. The study was reliant on young people self-selecting to participate in the interview. A Participant Information Sheet was provided for young people who were interested in being interviewed. Informed consent was obtained from participants and their parents. The largest number of responses throughout our study came from students in Years 9 and 10.

The study did not seek to find a representative sample of participants across geographical areas, socioeconomic groups, gender, ethnicities or other demographics. We interviewed eight young people, three boys and five girls, in Years 9 and 10 of secondary school. Six students were from greater Sydney, one was from regional Victoria and one was from regional NSW. Five of the eight students were in public secondary schools and the other three in Catholic or independent schools. Two students attended single sex girls’ schools and while all students spoke English at home, for two of the students English was an additional language. While our participants had access to individual computers and stable internet, we contend that participants’ personal and economic backgrounds spanned a wide range.

Interviews lasting 30–45 min were conducted by Zoom during July–September 2020. Interviewing students retrospectively about their experiences of initial periods of school closure was an appropriate risk-mitigating strategy in volatile and difficult times. The corpus of data analysed for this article entails transcripts of eight semi-structured interviews. Questions addressed school and learning, life with friends and family, emotions, confidence and trust, online life, Covid-19 generally and the future. As a further risk-mitigation adjustment, we added a final question inviting young people to reflect on positive actions they had taken despite the adverse conditions, that is on personal agency and its impacts. A thematic content analysis (Saldaña, 2016) using an iterative approach was developed with initial analysis centred on identifying and coding participants’ views about their experience of lockdown, domains of social life and modes of sociality. Three interconnecting themes were generated through the analysis: agency, resilience and sociality. Reflections about schooling, learning, and lives on and off-line thread through the following sections.

## Agency

When learning activities are predetermined and highly prescriptive, there are fewer opportunities for students to engage in planning, decision making, and reflection. It is widely recognised that students are more motivated to learn when they can play an active role in choosing what and how they will learn (OECD, 2019). Agency, as we have noted, is significantly constrained by the practices of schooling as usual, and what limited opportunities there are for student voice to be heard are unevenly available and tend to be tokenistic. Most of the students we interviewed did not seem to have input into topics or tasks and most learning seemed to involve textbooks, worksheets and attendance at online lessons. Their teachers would have had to prioritise keeping up with the curriculum that had been planned before the sudden lockdown. The only reference to a responsive curriculum opportunity was Fred’s mention that in History, they were asked to make up a story or diary about how Coronavirus was affecting them but the press of other work, including additional work that his father had sourced from overseas, meant there wasn't enough time to do this History task. However, our participants did offer many examples of moments when they were able to exercise agency about more mundane but important aspects of their lockdown learning lives.

Learning to manage time and develop new routines helped them to take charge of their learning and other aspects of their lives. This strengthened their belief in their ability to manage amidst the uncertainty and frustration associated with Covid-19. Tilly described the positive effect on her health: “I’ve found it a lot easier to do exercise. When you’re at school … it’s hard to find motivation after like a long day at school to do something or get up early in the morning.” Despite this period of high stress and societal anxiety, participants like Meg were able to organise themselves and to complete their work independently: “I know that I can work independently, I was not sure I could. I don’t need as much help from mum and dad with assessments. Everyone is feeling the same”. Students were surprised and pleased, finding capacities for independence and personal goal-setting that they had not previously recognised in themselves.

The shift to online learning became a fundamental part of school life, which continued when students returned to school after the initial lockdowns. Many of the students described how digital platforms had facilitated their learning. Meg, for example, also talked about this as facilitating new insights into how learning can happen: “We’ve discovered that online and zoom and facetiming can work and we are now using it.” While students were proud of their capacities to complete work to their own schedules, real-time lessons are important to facilitate peer interaction and help student motivation (Eivers et al., 2020; Schleicher, 2020). Many students missed the ease of clarification afforded by face-to-face classroom interaction. Learning is a social process where understanding is contingent on, and enhanced by, learning alongside others. Kai found learning “more difficult without your classmates, without cooperative working, [without] explanations from teachers.” However, as Meg also noted, teaching is more dynamic and relational than merely presenting information. In the following comment she seems to equate a didactic mode of online delivery with the ‘absence’ of the teacher: “The absence of the teacher was a massive struggle; no interaction happens when the teacher is explaining on a screen.” To Meg, what is most important is interactivity, and personalised attention.

In terms of student agency and control over their learning, the limits of autonomy may come when what they are learning exceeds what they already know, or what they feel they are capable of. Maths was particularly concerning. While asynchronous support was available, it was not necessarily efficient. As Tilly noted, “now it takes a whole email just to ask one question.” Agency was evident where students used their own initiative to seek help. Tilly, who experienced school closures for most of the year, contacted her Maths teacher from the previous year, who had invited students to stay in touch, for a Webex meeting. Not all students would have this confidence, and not all teachers would welcome it. However, this reinforced Tilly’s sense of control over her learning in a volatile time. Students who are able to set and pursue their own learning goals may also be more proactive in supporting their peers (Waite, 2020). Students formed digital networks to support each other’s learning, and made individual approaches to each other at points of need. Peers often contacted Tilly, for example, for help with schoolwork during the extended lockdown: “Lots of my friends, actually, especially at the start, were calling me a couple times every day for help … they were struggling a bit at home.”

Student agency and sense of control over their learning is contingent on how they feel they are progressing. Young people found the period of remote learning empowering when they felt that their skills were improving, despite the change in circumstances. Improvements in one domain of knowledge could extend beyond that domain. For example, while Fred stressed that his reading had improved, the impact is evident more broadly: “Right now I’ve improved. My reading skill is improving also … my learning at school is improving well too. And yeah, I’m starting to improve that and I’m happy.” For Kai, who identified coding as the domain of knowledge that progressed most during the lockdown, his sense of competency extended to broader aspects of his life, and was again related to increased autonomy and control:

I can schedule everything and what to do at what time. I learned more of that. Yeah, so I have more goals like getting a job and making more friends and learning how to be more social and learn more social skills. (Kai)

Our participants also identified many problems with online learning. These were usually when work set was repetitive and tedious, or when it offered no agency or choice for them as learners. Among our participants, Allan’s teachers relied most often on textbooks or photocopies distributed via Google classroom:

They’d say do this and then that’s your week’s worth… And then there’d be like instructions, like, do question one through this and then mark your answers. And then that’s it…I did all of it. But I could finish it all on a Monday and it would take like, an hour or so. And then I’d be done for the week. (Allan)

Australian schools used various platforms including Google Classroom, Google Meets, Microsoft Teams, Canvas, Zoom and others, but within timetabled online lessons pedagogies were often unengaging. Most of our participants mentioned worksheets as tedious or inefficient features of their lessons:

We would have the Google Meet at the start. And then they’d say, complete this worksheet... I would finish it within like half an hour in an 85-minute lesson… I actually ran out of work to do. When I was in lessons, I would be sitting there and I would be already done. (Anne)

Students became bored with the same approaches within their lessons, and with logging on to the same platforms. Allen said this made it “hard to be motivated” while Shelby found that learning became “a bit repetitive”. Although some students have adapted to learning online, concerns have been raised about the over-use of technology and increased screen time (Larcher et al., 2020). Our participants felt this fatigue as well, with Shelby finding it at times “overwhelming”. For Meg, it was “difficult” to “just stare at a screen around eight hours a day”, while Kai did not “enjoy…being in front of a screen for so many hours of the day”. Although our participants appreciated their abilities to keep up with their work and felt they had more control over schedules, their accounts of online learning did not include any examples of independent inquiry, project-based learning or collaborative learning that might have been feasible. Nevertheless, the overall impression that these eight young people gave us about the term (or more) they spent online learning was that they had increased their independence and confidence, particularly when they had some control over their learning and most of them were surprised by this.

## Resilience

Despite the disruptions to their everyday lives, many young people exemplified resilience in their willingness to engage with their learning and each other in volatile conditions. They gave many examples of activities they took up to enhance their own wellbeing and the wellbeing of others, and therefore, increasing their abilities to cope with the adverse conditions. Care for others, as much as care for the self, is crucial in this relational understanding of resilience. Kai collapsed these together in his description of how he stepped up in the crisis:

I can take care of myself. I can clean …and I can take care of my family and I can do more things to help them out… I definitely feel more connected and more, like we like care, and we interact more. (Kai)

Having a safe and secure home, and positive relationships with caring adults and peers helps young people develop their capacity to be resilient. In Fred’s family, the new evening ritual of reading the Bible together followed by a family movie was the “best thing” for him about the lockdown. For Tilly, whose parents went to work while she stayed home alone, having them come home for lunch was an important focus of her day. Anne’s family started a weekly cocktail night, during which they zoomed with relatives. Each student, in their varied contexts, found ways to connect differently with their families. While maintaining physical health was important for most of our participants, Fred linked this to his deepening connection with his father: “I was being trained by my dad most of the time and my mental health and physical health improved.”

Despite social isolation, young people took advantage of opportunities to be innovative and to try different approaches to deal with issues they encountered. This has led to some students finding creative ways to entertain themselves, as well as their friends and others around the world. The creativity was often generated in homes and driven by individuals, with artistic pursuits flourishing in unlikely spaces (Gannon et al. 2021). New spaces for pursuing creative passions were evident among our participants. Tilly had resumed her guitar lessons online: “I didn't do it the first time around because I thought it would just be too difficult. But it’s really not at all. It’s the same really. It’s so easy.” Anne saw the practice of creativity as part of a broader responsibility she felt to maintain and spread happiness in difficult times. She made sure to keep “doing things that I knew made me happy, to keep my happiness up and to make sure that I didn’t start to feel lonely or upset”. Keeping herself happy also reduced pressure on her family. Creative practices were central to her dynamic notion of happiness in motion:

Everyone inspires each other … if you saw someone else doing something that looked fun, like even on TikTok or the trends that people were doing [tie-dying, dancing videos] to entertain themselves, you see that and you go “Oh, I want to try that too”. And that’s just another way that happiness is spread. (Anne)

School closures and home confinement potentially have a negative effect on young people’s physical health and their social and psychological wellbeing (Wang et al., 2020). All our participants talked about missing the physical activities they would normally have accessed through school. Alyaa missed the social benefits of doing physical exercise at school with peers, where “it was just easier and a bit more fun with my classmates”. Our participants had lost opportunities for high level competitive basketball (Meg), dance (Alyaa), netball and football (Tilly) but when lockdowns eased they each found ways to meet with one or two friends for cycling, paddle-boarding, surfing, or walking in the park or neighbourhood. Recreational activities also brought families together. Alyaa’s family played more games, from board games through to playing cricket in the park. For her, “doing things that matter away from screens, [was] like we’ve started finding new ways to entertain ourselves together.”

Though resilience was not a specific focus in our interviews, their elaborations of what they missed and how they developed alternative coping strategies suggested the profoundly relational quality of resilience. Traces of the individualised subject responsible for managing their feelings in productive ways were evident when students talked of the need to keep themselves happy or healthy in order to manage stress, but their accounts suggest a socially responsible and socially embedded mode of resilience, where kinship and friendship are crucial social resources.

## Sociality

The profound sense of the social was shared through our participants’ accounts of their experiences of the isolation forced by Covid-19 and links with the ways they expressed and understood agency and the modes of resilience they seemed to enact. Schools are important in providing students with a sense of belonging and social contact, and supporting social and emotional wellbeing (Brown et al., 2020). However, only one of our participants talked about a school-led initiative of after-school activities intended as a “kind of an upkeep of the community” (Anne). Much more important to them were the online connections they maintained themselves. These “networked publics” have always been crucial components of teenagers’ broader social interactions, and sources of anxiety for the adults around them (Boyd, 2014). During school lockdowns, being online almost all the time became essential to their capacity to learn, but it was also a necessary condition of their capacity to cope emotionally.

This entailed flipping from the common-sense of young people as ‘at risk’ as inexperienced or vulnerable users of technology, to recognition of “young + digital” as a position from which young people enact agency and online engagement brings opportunities and benefits (Third et al., 2019, p. 57). Already adept users of social media, our participants reported increased and purposeful engagement with a wider range of platforms than before and had a very clear sense of their positive impacts and the agentive possibilities that emerged from co-production and experimentation with new platforms.

Maintaining social connections was their major preoccupation away from school. Shelby described how communication was vital for overcoming loneliness: “Keeping contact with everyone that you would normally see was really positive … created a better mindset because you didn’t feel as alone.” All participants used social media platforms to stay connected with their friends and classmates. Using photo-sharing apps like Snapchat with his friends meant that Fred could immediately see “how they are looking and it makes you understand that they’re ok and everything”. Our participants also conceded that they engaged with wider publics for, in Anne’s terms, “fun and inspiration”. Uploading TikTok dance challenges was a way of bringing happiness, not just to oneself but to the countless others in lockdown who might see it too. Alert to adults’ concerns about their privacy and online safety, our participants were quick to reassure us that they mostly engaged with people they already knew, and that this had completely positive impacts on them and on others. Staying connected took up a large amount of time, but they saw this as a worthwhile and necessary investment in wellbeing, this dynamic action often enhanced relationships.

Paradoxically, separation from friendship groups seemed to bring young people closer to each other. Shelby noted that “obviously the distance is going to make your friendship stronger” because friends were more inclined to confide in each other, which made the relationships both closer and stronger. Likewise, Alyaa said “it kind of brought us closer together strangely”. Kai extended his network by getting to know people better as they shared favourite movies and foods, finding out what they had in common. He described this as follows: “I am possibly better friends as we interact frequently, we spend more time online and talking and interacting.” Among Anne’s friends, peer support was particularly important when “one friend had an eating disorder during Covid and we were all supporting her. Tilly noticed that online connecting was more prevalent among her friends who were girls, as in her experience “boys don’t really Facetime or anything like that”.

Beyond their friends and classmates, the lockdown period also provided opportunities for some students to connect with people they previously had less contact with. For Kai this included young people outside his immediate friendship circle and extended family members overseas. Kai talked more often with “family back in Vietnam, grandma, grandparents, old friends and my cousin”. Fred also talked frequently with family in Africa. Shelby found this was a time to get to know her cousins overseas much better. She said “It was nice to actually talk to them more often, instead of being so rushed around and just actually having the time to talk to people.”

For our participants this new sense of time extended into family interactions and sociality within the home. For young people who previously spent most of their time apart from their families at school or engaging in extra-curricular and sporting activities, the stay-at-home restrictions provided opportunities for families to engage in the “ordinary magic” of everyday life by going for walks and preparing meals together (Dvorsky et al., 2020, p. 1829). Such changes in the frequency and type of family-based activities during the lockdown period can enhance family cohesiveness and mental health (Courtney et al., 2020). Meg was getting along much better now with her father. Alyaa expected to sustain this closer relationship by being “more proactive” in spending time with family in the future. Tilly described how changing practices of sociality within her home were the best things about the extended lockdown:

I think being around my mum and my family a lot more … I’d be at school all day. And then I’d probably come home and spend a fair bit of time in my room. Now I’m a bit more social … because I’m home a lot more and so are they (Tilly).

Our research challenges the singular narrative of the pandemic that characterises students as powerless victims without agency at a time of crisis and suggests that increased agency, resilience and sociality are also part of these young people’s experiences. Similarly, in the Danish study, young people actively sought out each other, communicated through social media, cultivated their creative capacities and escaped with friends to enjoy neighbourhood activities (Hansen et al., 2020). Like their Danish counterparts, who controlled their learning and supported their own and others mental health and wellbeing, our young Australian participants had a strong sense of responsibility and some confidence in their own capacities to get through this time.

At the time of our interviews in late 2020, their intimations of how the future might unfold were characterised by uncertainty. Tilly said “I don’t think you’re ever really go back to how it was before”, and Kai has changed his views “on the way we live and the way we interact” Anne pointed out both strengths and inconsistencies in the Australian response: “I feel stronger than before that community is more important than individual during the pandemic. But government policies are kind of contradicting that.” Perhaps this reflects a sense of security and trust experienced by these young people at the local scale, tempered by uncertainty about how government at the highest national level was managing the crisis.

## Conclusion

The shift to online learning came with numerous challenges for the young people we interviewed: unengaging pedagogies (even on digital platforms), difficulties of being separated from friends and extended families, lack of ongoing teacher support, timely feedback and clarification of concepts. However, this study also shows that there were Covid-19 induced opportunities for the young people to show tremendous strength, endurance and adaptability. The young participants used familiar online tools and media in new ways to re-enact, adapt, and develop new patterns of action for schooling and socialising. School closures forced them to “think outside the box” (OECD, 2020a, p. 3) and while digital technology was at the centre of teaching and learning, the transition to at-home learning enabled them to have greater control over many aspects of their learning as a way of managing the disruption to their everyday lives. The Danish study similarly recommended the need for digital contact and communication to be mixed with analogue forms of interaction and presence going forward. So rather than teachers structuring how and when students learn, students showed initiative and the capacity to set goals, reflect, and act in responsible ways in order to bring about change (OECD, 2019).

Arundhati Roy speaks of the pandemic as a “portal” or “gateway between one world and the next” (2020). Her invitation to “break with the past and imagine [our] world anew” invites us to discard the forces that prevent us from having a prosperous and just future (Roy, 2020). Rather than understanding the pandemic as a catastrophe of interrupted learning, missed milestones and postponed exams, the young people we spoke to showed us that it was a time they used to deepen connections with family and friends, to take charge of their own learning and to make responsible and caring decisions for their own and the good of others, and the wider society. Young people are more adept at navigating digital everyday life than is usually recognised, including taking up opportunities to imagine and work towards better worlds (Third et al., 2019). A switch to more independent online learning supported these young people to transform and modify the learning space and the relationships associated with it. It changed their positions as meaning makers, and enabled them to envisage possible futures for a more inclusive and resilient post-Covid world. These qualities might be named, acknowledged, encouraged and cultivated more directly by their schools as they are the qualities that are empowering young people to get through these fractured times while also offering a feasible educational alternative to traditional ways of learning.

## Footnotes

1 Earliest school lockdowns occurred in early March 2020 with individual schools closing for short periods to deal with outbreaks among staff. By the end of March all state educational authorities had agreed to start Term 2 with online learning from April 7. Victorian state school students returned to school in two waves on May 26 (1 & 2, 11 & 12) and June 9 (3–10). As the long lockdown of the second wave began in Melbourne at the start of Term 3, on July 13 most students in Melbourne were ordered to continue online learning. By August 5 all students in the state were remote learning. This continued into Term 4 with staggered return to on-site learning, with all students back to on-site learning by 26 October. In NSW, after sporadic outbreaks the Premier announced on March 23 that all students who could study from home should stay at home in Term 2. All students in state schools returned to on-site learning on May 25. Sporadic closures for deep cleaning of schools after case detection have continued. As the Delta variation circulated in 2021, school lockdowns continued as a major strategy of containment meaning that all of these students would have experienced a second year of extended school lockdowns subsequent to our interviews with them. We hope that some of the qualities that they identified in the first year have helped them through this next phase of the long pandemic. As we finalise this article, schools are open and secondary students all have access to effective vaccinations.
