# Correction to: Sociality, resilience and agency: how did young Australians experience online learning during Covid-19?

**DOI:** 10.1007/s13384-022-00512-9

**Published:** 2022-04-04

**Authors:** Loshini Naidoo, Jacqueline D’warte, Susanne Gannon, Rachael Jacobs

**Affiliations:** 1 School of Education, Western Sydney University, Second Ave, Kingswood, 2747 Australia; 2 School of Education, Western Sydney University, Bankstown Campus, Milperra, 2214 Australia

## Correction to: The Australian Educational Researcher https://doi.org/10.1007/s13384-021-00500-5

In the original publication of the article, under the heading “Young people, agency, resilience, sociality and learning in pandemic times” on the 12th line, the sentence “(REMOVED FOR BLIND REVIEW)” was incorrectly provided. It has now been removed.

The original article has been corrected.

